# The Importance of the Secure Base Effect for Domestic Dogs – Evidence from a Manipulative Problem-Solving Task

**DOI:** 10.1371/journal.pone.0065296

**Published:** 2013-05-29

**Authors:** Lisa Horn, Ludwig Huber, Friederike Range

**Affiliations:** 1 Department of Cognitive Biology, University of Vienna, Vienna, Austria; 2 Clever Dog Lab Society, Vienna, Austria; 3 Messerli Research Institute, University of Veterinary Medicine Vienna, Medical University Vienna, University of Vienna, Vienna, Austria; University of Arizona, United States of America

## Abstract

**Background:**

It has been suggested that dogs display a secure base effect similar to that found in human children (i.e., using the owner as a secure base for interacting with the environment). In children, this effect influences their daily lives and importantly also their performance in cognitive testing. Here, we investigate the importance of the secure base effect for dogs in a problem-solving task.

**Methodology/Principal Findings:**

Using a manipulative task, we tested dogs in three conditions, in which we varied the owner's presence and behavior (Experiment 1: “Absent owner”, “Silent owner”, “Encouraging owner”) and in one additional condition, in which the owner was replaced by an unfamiliar human (Experiment 2: “Replaced owner”). We found that the dogs' duration of manipulating the apparatus was longer when their owner was present than absent, irrespective of the owner's behavior. The presence of an unfamiliar human however did not increase their manipulation. Furthermore, the reduced manipulation during the absence of the owner was not correlated with the dog's degree of separation distress scored in a preceding attachment experiment.

**Conclusions/Significance:**

Our study is the first to provide evidence for an owner-specific secure base effect in dogs that extends from attachment tests to other areas of dogs' lives and also manifests itself in cognitive testing – thereby confirming the remarkable similarity between the secure base effect in dogs and in human children. These results also have important implications for behavioral testing in dogs, because the presence or absence of the owner during a test situation might substantially influence dogs' motivation and therefore the outcome of the test.

## Introduction

Based on ethological principles, Bowlby [1], [2] formulated the theory that for the survival of infants in humans as well as in many non-human animal species it is essential that infants develop a strong affectional bond with their primary caregiver – usually the mother. Separation from the attachment figure activates the infant's attachment system, which aims at restoring and maintaining proximity with this specific individual [1]. Four particular behavioral components can be used to discriminate a true attachment bond from other affectional bonds [3]: a) staying near to and resisting separation from the attachment figure (proximity maintenance), b) feeling distress upon involuntary separation from the attachment figure (separation distress), c) using the attachment figure as a base for exploring the environment free of anxiety (secure base), d) seeking out the attachment figure for contact and assurance in times of emotional distress (safe haven). Ainsworth [4] argued that the secure base effect was the most important component of the attachment system, because it is crucial for balancing the maturing infants' exploration of the world with maintaining proximity to the caregiver. Although Bowlby's original attachment theory had been developed in regard to human children, the same behavioral components have been found in infant-caregiver relationships in many bird and mammal species (e.g., chicken, macaques, and dogs; for a review see [5]).

Domestic dogs have been closely associated with humans for about 15,000 years [6] and are so well adapted to their niche in the human society that in many cases the owner has replaced conspecifics as the main social partner. This unique relationship between adult dogs and their human owners bears a remarkable resemblance to an infant attachment bond: dogs are dependent on human care and their behavior seems specifically geared to engage their owners' care-giving system [7], [8]. Given the broad comparative framework of Bowlby's original theories, several researchers have used attachment concepts and methodology to investigate whether the dog-human relationship conforms to the characteristics of an attachment bond. For that purpose most researchers used an experimental procedure developed for testing human children by Ainsworth and Wittig [9] – the Ainsworth Strange Situation Test (ASST). In this test children are confronted with an unfamiliar setting, an unfamiliar person entering the room and two brief separations from the attachment figure in a fixed sequence. This mildly stressful setting is geared to activate the child's attachment system in order to measure each of its behavioral components separately (e.g., crying at the exit of the parent (separation distress), exploring and playing more in the presence of the parent than in the absence (secure base)). Using a modified version adapted for testing adult dogs with their human owners, two studies found clear evidence for proximity seeking and separation distress in the dogs [10], [11]. However, although the authors found more exploration in the presence of the owner than in the subsequent absence of the owner, they could not unequivocally attribute this to a secure base effect due to a strong confound with the sequence effects inherent to the procedure [11]. To control for this factor, Palmer and Custance [12] carried out a counterbalanced version of the ASST and found indications for a secure base effect independently from the sequence. However, according to attachment theory, the secure base effect should not only be evident in the ASST, but should influence most of the individual's interactions with the environment. In line with this, Matas et al. [13] showed that in human children the secure base effect had an influence on their performance in an experimental problem-solving task. Their study revealed that children, who were able to use their mother as a secure base for exploring the environment, were also more persistent and enthusiastic while solving the task than children for whom the mother was no secure base. Since the dog has emerged as a model species for behavioral and cognitive research in recent years, it is vital to understand whether the attachment to their owners – particularly the secure base effect – is also relevant for their performance in cognitive tasks as it has been shown for human children [13].

The aim of our study was to investigate the importance of the secure base effect for dogs in a behavioral test situation. In children the secure base effect is mostly investigated by comparing their motivation to play in the caregiver's presence and absence [9]. However, while children typically spend long periods with solitary play, most dogs do not. Therefore, we used a problem-solving task, which the dogs were motivated to carry out for a long time (i.e. manipulating an apparatus in order to obtain a food reward). In Experiment 1 we tested whether the presence or absence of the owner would influence dogs' motivation to manipulate an apparatus. We predicted that if the owner acted as a secure base for the dog, the dog's performance should be poorer in the owner's absence (Condition “Absent owner”). To control for the possibility that the difference in performance was brought about by the absence of behavioral cues from the owners, each dog was tested in two different conditions with the owner present: a) the owner was blindfolded and did not interact with the dog (Condition “Silent owner”) and b) the owner was allowed to encourage the dog verbally (Condition “Encouraging owner”). To further examine the effects of dogs' general distress during separation from the owner, all dogs were independently tested in a shortened version of the ASST and their degree of separation distress was compared to their performance in the task. In Experiment 2 of this study, we further investigated whether the secure base effect was specific to the owner, which would be predicted if the relationship resembled an infant-caregiver relationship, or whether it would extend to an unfamiliar human (Condition “Replaced owner”).

## Experiment 1

### Materials and Methods

#### Ethics Statement

Owners gave their written consent for participating in behavioral studies with their dogs when entering the database of volunteer participants of the Family Dog Research Program (http://kutyaetologia.elte.hu) at the Department of Ethology of the Eötvös Loránd University in Budapest, Hungary. Prior to the experiments reported in this manuscript, the owners were informed about the details of the procedures of each experiment and were given the possibility to withdraw from participation. No special permission for use of dogs in non-invasive studies is required in Hungary. The relevant committee that allows conducting research without special permissions regarding animals is the University Institutional Animal Care and Use Committee (UIACUC, Eötvös Loránd University, Hungary). Since the owners were only required to interact with their dogs in their usual manner during the experiments and their behavior was not coded or analyzed, no approval for human experimentation was obtained. The participants in the video clips that are part of the supporting information have given written informed consent, as outlined in the PLOS consent form, to publication of themselves.

#### Participants

Twenty-two dogs were recruited from a database of volunteer participants of the Family Dog Research Program at the Department of Ethology of the Eötvös Loránd University in Budapest, Hungary. Only dogs living permanently in the owner's household as pets were selected and all dogs had at least basic obedience training. All dogs were highly food-motivated. Two dogs had to be excluded because they failed the pre-test (see procedures section below). Therefore, 20 dogs (12M/8F; mean age ± SD = 2.7±2.36 years) completed the experiment. The sample consisted of 14 purebred dogs from three different FCI (Fédération Cynologique Internationale) breed groups (Sheepdogs: N = 9; Toy dogs: N = 3; Primitive types: N = 2) and 6 mixed-breed dogs.

#### Experimental Design

Dogs first had to pass a pre-test to ensure that they were motivated to manipulate an apparatus to get food and that they were willing to consume food even in the absence of the owner. Dogs that passed the pre-test by taking food in the absence of the owner progressed to the test phase. In the test phase, we used three different test conditions, in which we varied the presence of the owner and the owner's behavior:

- *Condition “Absent owner” (cAO)*: The owner is not present in the experimental room during the trial.
- *Condition “Silent owner” (cSO)*: The owner is present in the experimental room during the trial, but remains silent.
- *Condition “Encouraging owner” (cEO)*: The owner is present in the experimental room during the trial and is encouraging the dog verbally.

We used a within-subject design so that each dog received one trial in each of the three test conditions. The sequence of the conditions was counterbalanced across dogs.

#### Apparatuses

**Figure 1:**
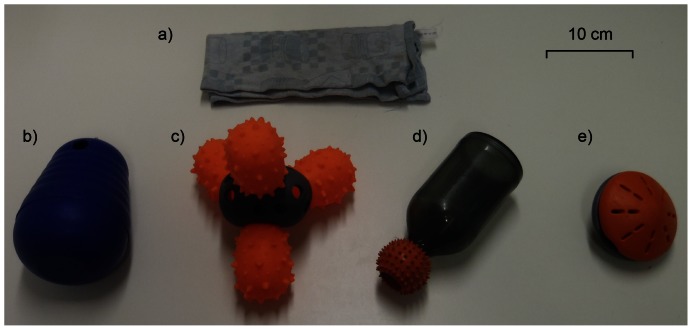
Apparatuses used in the experiment. a) Folded cotton towel used in the pre-test, b) Nina Ottosson^©^ Dog Pyramid (aDP), c) Hunter^©^ Snack Cactus (aSC), d) Hunter^©^ Snack Bottle (aSB), e) the smaller version of the Hunter^©^ Rolling Snack (aRS).

For the pre-test we used a folded cotton towel (13 cm×29 cm×2 cm) under which the food reward was placed. As apparatuses for the test we used four types of commercial interactive dog toys, which could be filled with food rewards: Nina Ottosson^©^ Dog Pyramid (aDP, 13 cm×13 cm×17 cm), Hunter^©^ Snack Bottle (aSB, 9 cm×20 cm×9 cm), Hunter^©^ Snack Cactus (aSC, 20 cm×20 cm×20 cm), and Hunter^©^ Rolling Snack (aRS, Figure 1). The latter toy was available in a smaller (10 cm×10 cm×7 cm) and a larger version (13 cm×13 cm×10 cm) and thus the size was adjusted to the size of the dog. All toys had to be manipulated persistently with either the paw or the muzzle to receive the food rewards placed inside. The food rewards were of high quality (i.e., dog sausage) and consisted of 5 pieces per trial. Before the experiment started we asked owners whether their dogs had already interacted with toys that were the same or similar to any of the four toys presented. For each dog we selected three toys that were unknown to the dog. Otherwise toys were selected randomly.

#### Experimental Set-up

**Figure 2:**
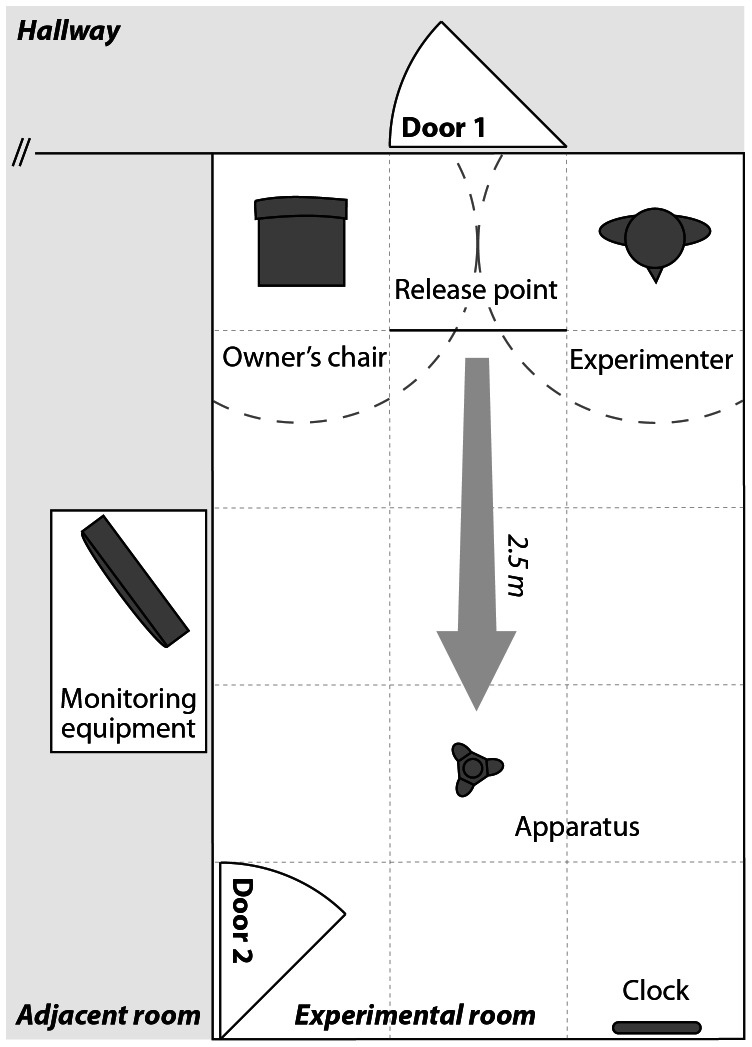
Schematic representation of the experimental set-up. The experimental room was equipped with two doors, one connecting it to the hallway (Door 1) and one connecting it to an adjacent room with monitoring and recording equipment (Door 2). At the beginning of each trial, the dog was released by the experimenter from the release point, which was 2.5 m away from the apparatus. During each trial the experimenter stood on the right side of door 1 – timing the trial with a clock on the opposite wall. The owner either sat on the designated chair on the left side of door 1 or was in the adjacent room – depending on the pre-test trial or the condition of the test trial. The dashed lines indicate the floor markings around the owner's chair and the experimenter's position, which were used for later video coding.

The experiments were carried out between February and June 2010 in a quiet experimental room (3 m×5 m) at the department. The room was equipped with two doors: door 1 could be used to enter the experimental room from the hallway, door 2 led to an adjacent room. A chair for the owner was placed on the left side of door 1. The experimenter was positioned on the right side of door 1 during the pre-test and test trials. Tape markings on the floor indicated a circular area (r = 1 m) around the owner's chair and the experimenter's position, respectively. A clock on the opposite wall was used by the experimenter to time the trials. A line on the floor marked the dog's release point. The apparatus was placed 2.5 m away from this line. The room was equipped with four cameras linked to monitoring and recording equipment in the adjacent room (Figure 2).

#### Procedures

All dogs participating in this experiment had previously been tested in a modified version of the ASST aimed at characterizing their relationship with their owners. This test consisted of several episodes in which either the owner, an unfamiliar human or both were present in the room with the dog. The test also comprised two separation episodes, which allowed us to assign a score of separation-related behavior (SRB) to every dog (see File S1 for a detailed description).

In the main experiment, the general procedure was the same for each trial of both the pre-test and the test phase. Before each trial, the dog always observed the handling of the food reward (pre-test trials, test trials) and/or the baiting of the apparatus (test trials) in the hallway outside the experimental room. The experimenter then entered the room through door 1 and placed the food reward or the apparatus on the designated position (see Figure 2). After that, the experimenter returned to the hallway. Right before each trial the owner entered the experimental room through door 1 while the dog waited in the hallway with the experimenter. The owner then took position depending on the pre-test trial or the condition of the test trial. In each trial, the owner had to wear dark sunglasses (either opaque or normal), which allowed us to manipulate the owner's visual access to the dog's actions depending on the condition. After waiting for 10 s, the experimenter entered the room together with the dog on a leash. She directed the dog to the line indicating the release point and released it with one command (i.e. “You can go, it's yours!”). Then she took her position next to the door. During the trial the experimenter never looked at the actions of the dog but kept looking at the clock on the opposite wall. After a pre-defined time (see detailed descriptions below) the trial ended and the experimenter called the dog back, put it on leash and walked it out of the experimental room. The owner waited for 10 s and then also left the room through door 1. There was a break of 5 to 10 min between trials.

Pre-test phase – The pre-test phase consisted of four trials that were administered in a fixed order:

- *Trial 1*: Owner present, food reward on the floor
- *Trial 2*: Owner present, food reward under the towel
- *Trial 3*: Owner absent, food reward on the floor
- *Trial 4*: Owner absent, food reward under the towel

In the first two trials of the pre-test phase the owner sat down on the chair and put on opaque sunglasses. The food had either been placed in the location of the apparatus directly on the floor (Trial 1) or under the towel (Trial 2). Trial 1 ended when the dog had consumed all five pieces of food or after a maximum of one minute. Since it was not possible for the experimenter to assess whether the dog had retrieved all pieces of food from underneath the towel, trial 2 ended after exactly one minute. In trial 3 and trial 4 the owner – after first entering the experimental room though door 1 – left the room through door 2 and remained in the adjacent room during the trial. The rest of the procedure was the same as in trial 1 and trial 2, respectively. In the adjacent room the owner could observe the events in the experimental room via monitoring equipment. The owner therefore knew when the trial was over and he or she had to leave this room and exit the experimental room through door 1 again.

Two dogs were not motivated to consume any piece of food from the floor in the absence of the owner (Trial 3) and could therefore not proceed to the test phase. Two further dogs failed to retrieve any piece of food from under the towel in the absence of the owner (Trial 4). However, these dogs were motivated to retrieve the food and actively manipulated the towel during this trial. Therefore we decided to include them in the test phase.

Test phase – In the test phase, we administered three trials, each of which lasted 5 min.

In a trial of the condition “Absent owner” (cAO) the owner left the room in the same way as in the trials 3 and 4 of the pre-test phase (see Movie S1). In a trial of the condition “Silent owner” (cSO) the owner sat down on the chair, put on opaque sunglasses and remained silent and passive throughout the trial (see Movie S2). In a trial of the condition “Encouraging owner” (cEO) the owner sat down on the chair, put on normal sunglasses and was allowed to encourage the dog verbally and to point at the apparatus throughout the trial. The owner had to remain seated on the chair but was allowed to pet the dog when it came close. However, the owner was not allowed to touch the apparatus (see Movie S3). The sequence of conditions was counterbalanced across dogs.

#### Data Analysis

All experimental sessions were videotaped for later behavioral coding with Solomon Coder beta (©2006–2011 András Péter). All statistical analyses were carried out with SPSS Statistics 17.0.0 (©2008 SPSS Inc.).

Since we were interested in the dogs' motivation to manipulate and not in potential individual differences in their ability to retrieve the food, we coded their duration of manipulation in the test trials and did not analyze their success. “Manipulating” was recorded continuously whenever the dog was interacting with the apparatus with its muzzle or paw. Additionally, “staying close to the owner” and “staying close to the experimenter” was recorded continuously whenever the dog was with at least one paw and the head within the area marked by the circle around each respective person. A second coder blind to the aim of the experiment and to the experimental conditions coded 20% of the videos of the test trials and Cronbach's alpha was calculated as a measure of inter-observer reliability. Cronbach's alpha was greater than α = 0.9 for all behavioral variables.

A linear mixed model (LMM) with main effects and two-way interactions was used to investigate the effect of the factors “sequence of conditions” (1^st^, 2^nd^, 3^rd^), “type of apparatus” (aDP, aSB, aSC, aRS), and “condition” (cAO, cSO, cEO) on the variable “duration of manipulation”. To investigate whether the duration that dogs spent manipulating the apparatus when the owner was absent (cAO) was correlated with the degree of SRB scored during the ASST, we used Spearman's rank correlation. To control for individual variation in general manipulation durations, we calculated a “relative duration of manipulation in cAO” by dividing the absolute duration of manipulation in cAO by the dog's average duration of manipulation across all three trials and correlated this score with the dog's SRB score.

Additionally we calculated two separate LMMs (main effects, two-way interactions) where we investigated the effects of the same factors as above on the variable “duration spent in the proximity of the owner” in those conditions where the owner was present in the room (cSO, cEO) and on the variable “duration spent in the proximity of the experimenter” in all three conditions.

Analyses of the residuals confirmed normal distribution for all variables in the three LMMs. Post-hoc comparisons of estimated marginal means (EM means) were carried out with LSD confidence interval adjustment.

### Results

**Figure 3:**
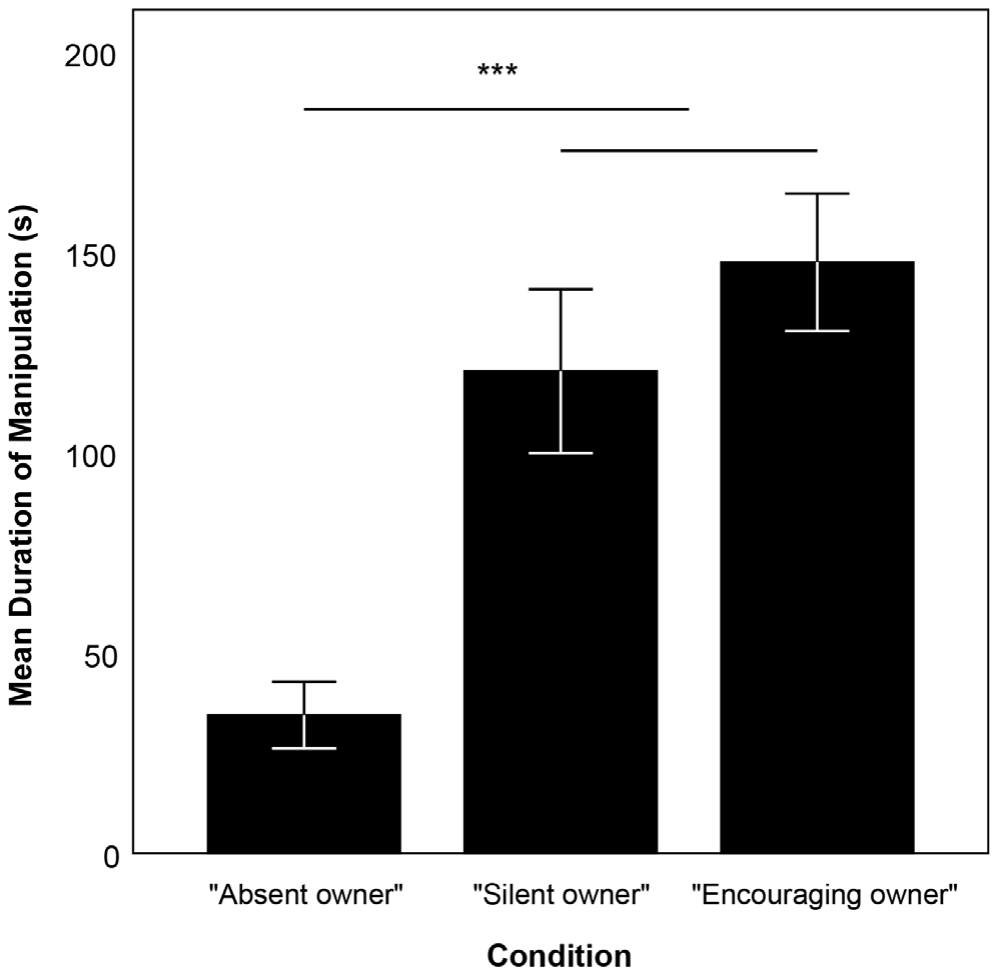
Duration of manipulating the apparatus – **Experiment 1**. Mean duration of manipulating the apparatus in the conditions *“Absent owner”* (cAO), *“Silent owner”* (cSO), and *“Encouraging owner”* (cEO). Shown are mean ± s.e.m. *** represents P≤0.001.

We found that the condition in which the dogs were tested had a highly significant effect on how long the dogs manipulated the apparatus (LMM, N = 20, F_2,36_ = 12.478, P≤0.001, Figure 3). The dogs manipulated the apparatus shorter when the owner was absent (EM means, cAO-cSO: P = 0.001, cAO-cEO: P≤0.001), while there was no significant difference between the two conditions when the owner was present (EM means, cSO-cEO: P = 0.540). Neither the sequence nor the type of apparatus, nor any of the interactions had an effect on how long the dogs continued to manipulate the apparatus (full results of the LMM can be seen in File S1). When analyzing the correlation between the dogs' relative duration of manipulating when the owner was absent and their SRB score assigned in the ASST, we found no negative correlation (Spearman's rank correlation, N = 20, ρ = −0.140, P = 0.557), indicating that the effect observed in the LMM was not only present in those dogs with strong separation distress but in all dogs of the sample.

**Figure 4:**
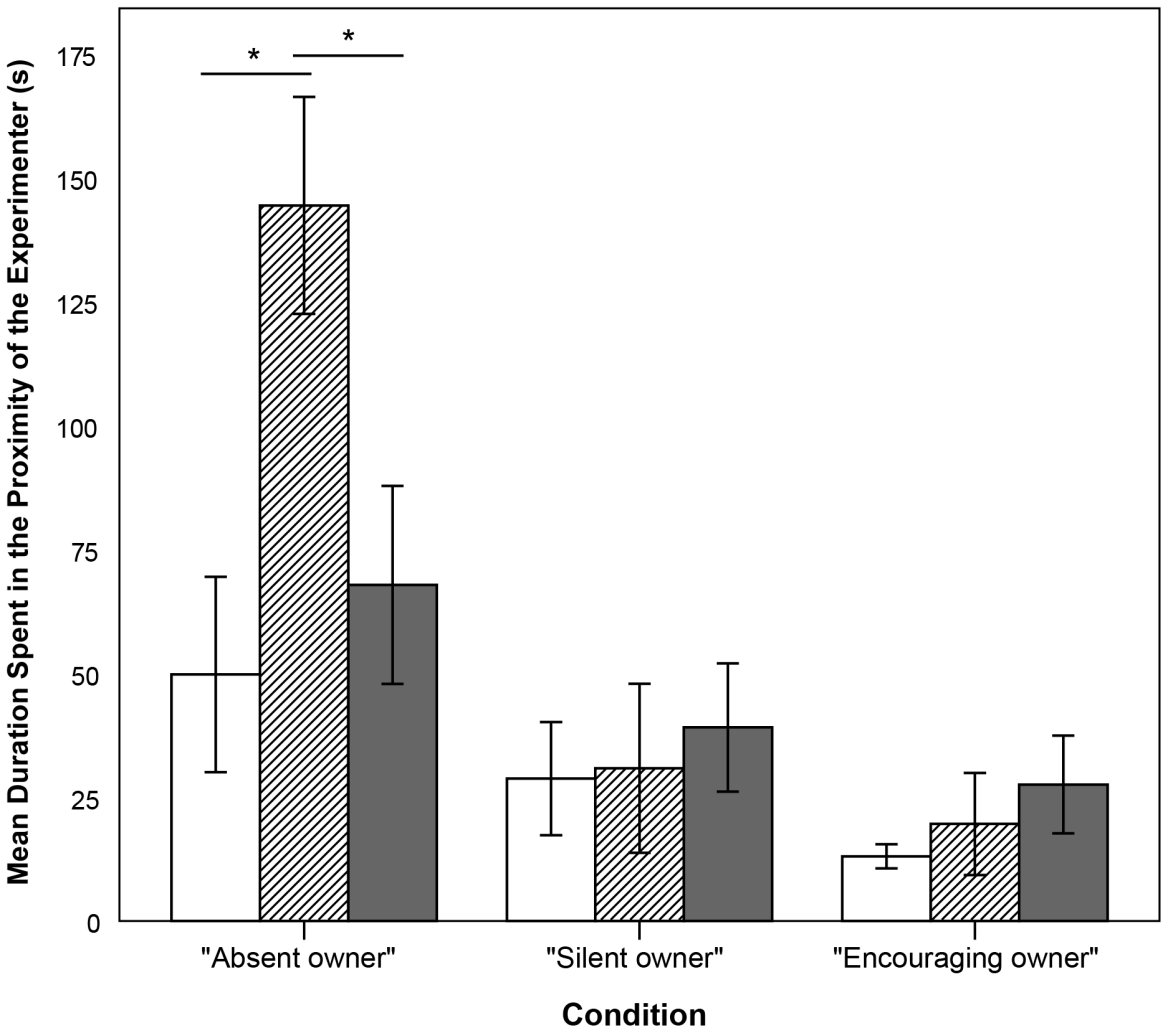
Duration spent in the proximity of the experimenter – **Experiment 1** **.** Mean duration spent in the proximity of the experimenter in the conditions *“Absent owner”* (cAO), *“Silent owner”* (cSO), and *“Encouraging owner”* (cEO). White bars represent the 1^st^ trials, striped bars represent the 2^nd^ trials, grey bars represent the 3^rd^ trials. Shown are mean ± s.e.m. * represents P≤0.05.

When investigating the time that the dogs spent in proximity of the two humans we found that the dogs spent overall equal amounts of time close to their owner in both conditions when the owner was present. Also, the sequence and the type of apparatus had no effect on this behavior (full results of the LMM can be seen in File S1). However, when looking at the duration that the dogs spent close to the experimenter, we found a highly significant effect of the condition. When the owner was absent the dogs spent more time close to the experimenter than in both conditions when the owner was present (LMM, N = 20, F_2,36_ = 17.221, P≤0.001; EM means, cAO-cSO: P≤0.001, cAO-cEO: P≤0.001, cSO-cEO: P = 0.593, Figure 4). Further, there was a significant main effect of the sequence (LMM, F_2,36_ = 4.611, P = 0.016) and a significant interaction term between condition and sequence (LMM, F_4,36_ = 2.923, P = 0.034; full results of the LMM can be seen in File S1), indicating that the effect of sequence on the time spent close to the experimenter varied according to the test condition. We therefore split the data into the three conditions. We found that in the condition “Absent owner” the dogs spent significantly more time close to the experimenter when they received this condition in their second trial than when receiving it in either their first or their third trial (pairwise comparisons, Mann-Whitney U test, 1^st^–2^nd^: Z = 2.429, P = 0.015; 2^nd^–3^rd^: Z = −1.981, P = 0.048; 1^st^–3^rd^: Z = 1.000, P = 0.317). In the other two conditions there was no effect of sequence (Figure 4).

### Discussion

In Experiment 1 we found that in a problem-solving task the dogs' duration of manipulating an apparatus for retrieving food was shorter when their owner was absent than when the owner was present, irrespective of the owner's behavior when present in the room. This significant decrease in manipulation in the absence of the owner was not only evident in dogs with strong separation distress, since the duration of manipulation was not negatively correlated with the dogs' degree of separation-related behavior scored in the ASST. The dogs' proximity to their owner during the experiment did not depend on the owner's behavior, whereas the dogs spent most time in the proximity of the experimenter when the owner was absent – especially when they received this condition as their second trial.

The effect of reduced manipulation in the absence of the owner found in this experiment cannot be attributed to a lack of food motivation in the absence of the owner or to the surprise of not finding the owner in the experimental room because with the pre-test we made sure that all dogs were familiarized with the owner's potential absence from the room and that they were ready to consume food also when separated from the owner. Additionally, the decrease of manipulation cannot be attributed to a lack of behavioral cueing or encouragement from the absent owner. In the condition “Encouraging owner” the owner was constantly encouraging the dog to manipulate the apparatus, while in the condition “Silent owner” the owner could not see the actions of the dog due to being blindfolded and did not interact with the dog at all. Despite these substantial differences in the owner's cueing and encouragement, there was no significant effect on how long dogs persisted in manipulating the apparatus in these two conditions. Therefore, the only factor influencing the dogs' duration of manipulation was the presence of the owner.

In our study we also analyzed whether the dog's decreased duration of manipulation in the owner's absence was due to separation distress rather than the lack of security gained by the presence of the owner. In this case, dogs that experienced strong separation distress would have been expected to manipulate shorter than dogs that were not distressed by the owner's absence. However, since the dogs' duration of manipulation was not negatively correlated with their individual SRB score, we showed that the owner's absence did not affect the dogs differently. These results point to the owner's function as a secure base for the dogs, influencing their persistency to manipulate the apparatus in this cognitive task.

However, we also found that the dogs interacted with the experimenter and spent more time in her proximity in the absence of the owner, possibly indicating that the experimenter had the potential to provide social support to the dogs in this stressful situation. It is interesting that the effect of searching the experimenter's proximity was strongest when the dogs received the condition “Absent owner” as their second trial. This cannot be attributed to the stronger insecurity about the absence of the owner after a trial with the owner present in the room because in that case the same effect should have also been found in the third trial. Additionally, in the pre-test all dogs were familiarized with the potential absence of the owner and therefore it should not have been an unexpected event for the dogs. Although the observed effect was very robust with an equal variation across the dogs, our experiment does not allow us to draw final conclusions and therefore further research will be needed to explain this effect.

The fact that the experimenter also seemed able to provide some social support for the dogs raises the question whether the effect of increased manipulation in the presence of the owner was due to a secure base effect or whether any human can provide security to a dog. In the latter case, the difference we observed between the two conditions “Owner present” and “Owner absent” might be due to a greater security provided by two humans (i.e., owner and experimenter) than by one person (i.e., only experimenter). Therefore, in Experiment 2 we added a new condition, in which an unfamiliar human replaced the owner (Condition “Replaced owner”).

## Experiment 2

### Materials and Methods

#### Ethics Statement

Prior to the experiment, the owners were informed about the details of the procedures of this experiment and gave their written consent to participate with their dogs. The study was discussed and approved by the institutional ethics committee (Ethik- und Tierschutzkommission) of the Veterinary University Vienna in accordance with GSP guidelines and national legislation (Approval date: 21.08.2012). Since the owners were only required to interact with their dogs in their usual manner during the experiments and their behavior was not coded or analyzed, no approval for human experimentation was obtained. The participants in the video clips that are part of the supporting information have given written informed consent, as outlined in the PLOS consent form, to publication of themselves.

#### Participants

Thirty dogs were recruited from a database of volunteer participants of the Clever Dog Lab Society in Vienna, Austria, using the same criteria as in Experiment 1. Three dogs had to be excluded because they failed the pre-test, which was the same as in Experiment 1. For one dog the experiment had to be aborted after the first trial due to considerably high stress levels when interacting with the apparatuses. Therefore, 26 dogs (11M/15F; mean age ± SD = 3.6±2.44 years) completed the experiment. The sample consisted of 17 purebred dogs from three different FCI breed groups (Sheepdogs: N = 10; Toy dogs: N = 5; Primitive types: N = 2) and 9 mixed-breed dogs.

#### Experimental Design

Dogs first had to pass the same pre-test as in Experiment 1. In the test phase, we used the three test conditions from Experiment 1 and added one new condition, in which we replaced the owner with an unfamiliar human to control for the specificity of the secure base effect:

- *Condition “Absent owner” (cAO)*: The owner is not present in the experimental room during the trial.
- *Condition “Silent owner” (cSO)*: The owner is present in the experimental room during the trial, but remains silent.
- *Condition “Encouraging owner” (cEO)*: The owner is present in the experimental room during the trial and is encouraging the dog verbally.
- *Condition “Replaced owner” (cRO)*: An unfamiliar human of the same gender as the owner is present in the experimental room during the trial and remains silent.

We used a within-subject design so that each dog received one trial in each of the four test conditions. The sequence of the conditions was counterbalanced across dogs.

#### Apparatuses

The same apparatuses as in Experiment 1 were used in this experiment. The apparatuses were unknown to all of the dogs and the sequence of apparatuses and the condition in which they were used were counterbalanced across dogs.

#### Experimental Set-up

The experiments were carried out between August 2012 and March 2013 in a quiet experimental room (3 m×6 m) at the Clever Dog Lab. The set-up was the same as in Experiment 1 (see Figure 2).

#### Procedures

Pre-test phase – The procedure of the pre-test trials was the same as in Experiment 1. The only difference was that in trials 1 and 2 both the owner and the unfamiliar human were present in the room, whereas in trials 3 and 4 both of them left the experimental room and remained in the adjacent room during the trial.

Test phase – The three test conditions “Absent owner” (cAO), “Silent owner” (cSO), and “Encouraging owner” (cEO) were the same as in Experiment 1, with the only difference that also the unfamiliar human entered the experimental room together with the owner through door 1 and then left through door 2 and remained silent in the adjacent room during the trial. In the new test condition “Replaced owner” (cRO) the owner and the unfamiliar human entered the experimental room together. The owner then left the room through door 2 and remained silent in the adjacent room. The unfamiliar human sat down on the chair, put on opaque sunglasses and remained silent and passive throughout the trial (see Movie S4). After the trial ended and the experimenter had left the room together with the dog, also the owner and the unfamiliar human left the room through door 1. The sequence of conditions was counterbalanced across dogs.

#### Data Analysis

All experimental sessions were videotaped, coded with Solomon Coder beta (©2006–2011 András Péter), and analyzed with SPSS Statistics 17.0.0 (©2008 SPSS Inc.).

The same three behaviors as in Experiment 1 were coded continuously (i.e., “Manipulating”, “Staying close to the owner”, “Staying close to the experimenter”). A second coder blind to the aim of the experiment and to the experimental conditions coded 20% of the videos of the test trials and Cronbach's alpha was greater than α = 0.9 for all behavioral variables. Where possible, the variables were lg10-transformed in order to apply parametric statistics.

A linear mixed model (LMM) with main effects and two-way interactions was used to investigate the effect of the factors “sequence of conditions” (1^st^, 2^nd^, 3^rd^, 4^th^), “type of apparatus” (aDP, aSB, aSC, aRS), and “condition” (cAO, cRO, cSO, cEO) on the variable “duration of manipulation”. Additionally we calculated a separate LMM (main effects, two-way interactions) where we investigated the effects of the same factors as above on the variable “duration spent in the proximity of the experimenter”. Analyses of the residuals confirmed normal distribution for all variables in the LMMs. Post-hoc comparisons of estimated marginal means (EM means) were carried out with LSD confidence interval adjustment.

We compared the duration spent close to the silent owner (cSO) to the duration spent close to the unfamiliar human (cRO) with a Wilcoxon signed-rank test.

### Results

**Figure 5:**
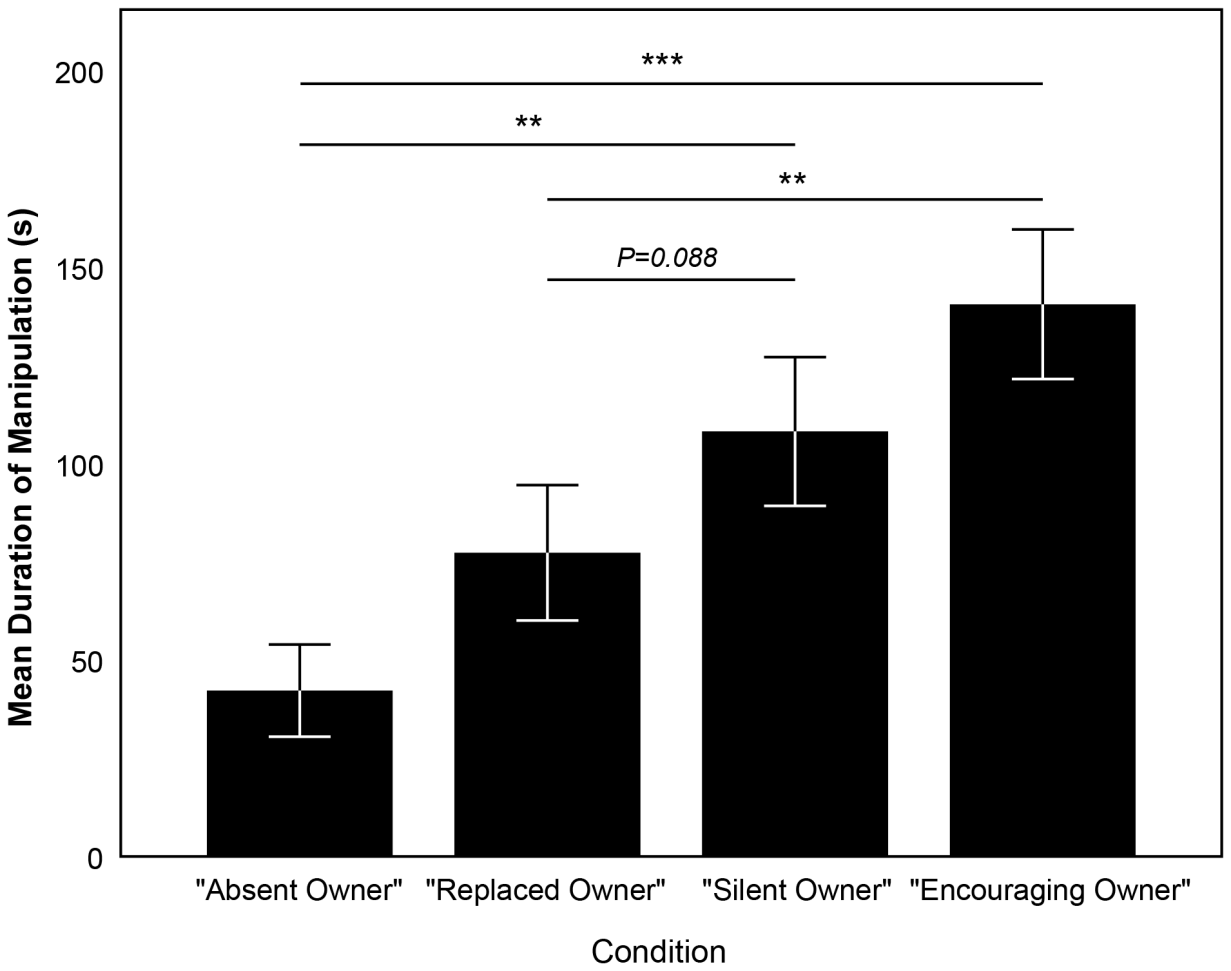
Duration of manipulating the apparatus – **Experiment 2** **.** Mean duration of manipulating the apparatus in the conditions *“Absent owner”* (cAO), *“Replaced owner”* (cRO), *“Silent owner”* (cSO), and *“Encouraging owner”* (cEO). Shown are mean ± s.e.m. *** represents P≤0.001, ** represents P≤0.01.

As in Experiment 1, the condition in which the dogs were tested had a significant effect on how long the dogs manipulated the apparatus (LMM, N = 26, F_3,67_ = 7.700, P≤0.001, Figure 5). The dogs manipulated the apparatus shorter when they were alone with the experimenter than in the two conditions when the owner was present (i.e., conditions “Silent owner” and “Encouraging owner”; EM means, cAO-cSO: P = 0.002, cAO-cEO: P≤0.001). The dogs also manipulated the apparatus shorter when the owner was replaced with an unfamiliar human than when the owner was present and encouraging the dog (EM means, cRO-cEO: P = 0.007). There was only a non-significant trend that dogs manipulated less when the owner was replaced than when the owner was present and silent (EM means, cRO-cSO: P = 0.088). There was no significant difference between the two conditions when the owner was present (EM means, cRO-cEO: P = 0.294) and the two conditions when the owner was absent (EM means, cRO-cEO: P = 0.137).

**Figure 6:**
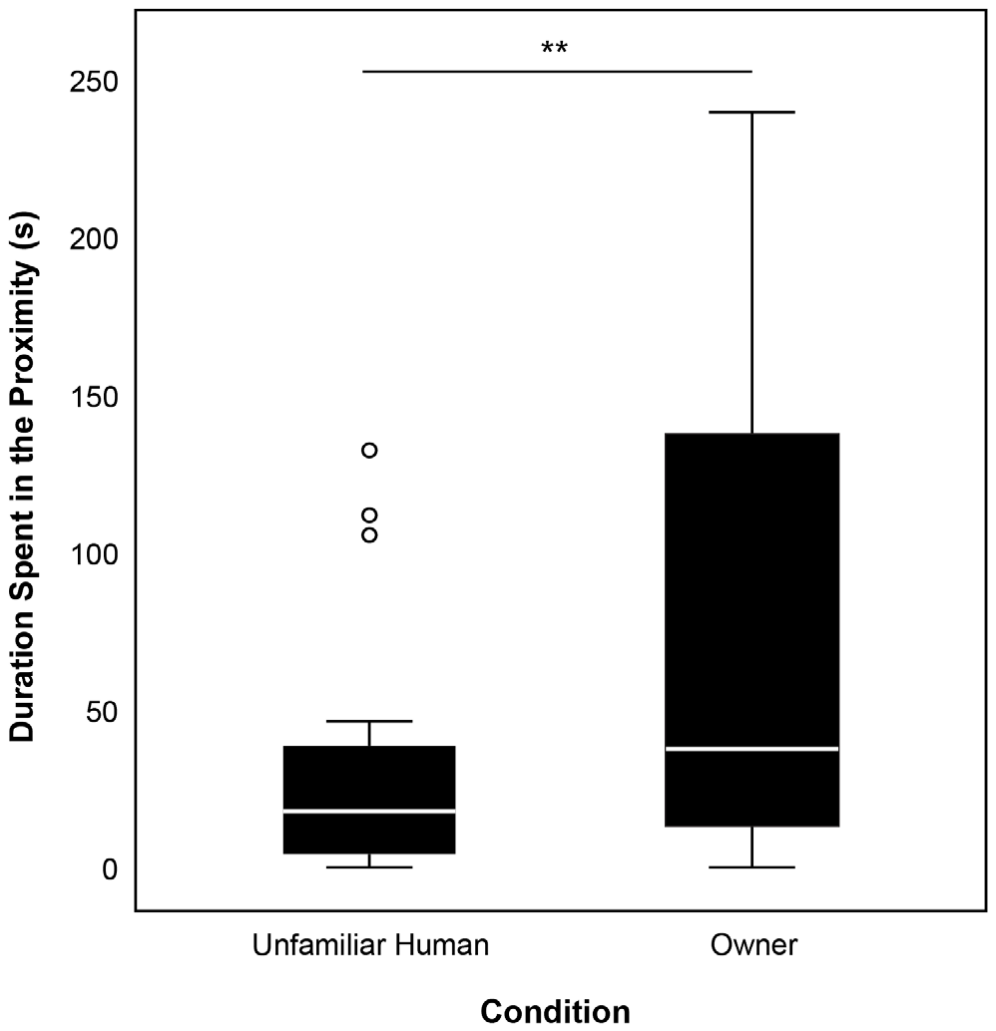
Duration spent in the proximity of the owner and the unfamiliar human – **Experiment 2** **.** Graph depicts box plots of duration spent in the proximity of the unfamiliar human (cRO) and of the owner (cSO), respectively. For each box plot, median values are indicated by the line within the box. The box represents 50% of the values (25th and 75th percentiles), with the upper bar representing the 90th percentile and the lower bar representing the 10th percentile. Circles indicate outliers. ** represents P≤0.01.

In this experiment dogs also differentiated between the apparatuses (LMM, N = 26, F_3,67_ = 5.509, P = 0.002). They manipulated the Hunter© Rolling Snack longer than all the other apparatuses (EM means, aRS-aDP: P≤0.001, aRS-aSC: P = 0.013, aRS-aSB: P = 0.004). Neither the sequence nor any of the interactions had an effect on how long the dogs continued to manipulate the apparatus (full results of the LMM can be seen in File S1). The dogs spent significantly more time close to the owner in the condition “Silent owner” than to the equally silent unfamiliar human in the condition “Replaced owner” (Wilcoxon signed-rank test, N = 26, W = 73.00, P = 0.009; Figure 6).

The dogs did not spend the same amount of time close to the experimenter in the different conditions (LMM, N = 26, F_3,67_ = 8.257, P≤0.001). As in Experiment 1, the dogs spent more time close to the experimenter when they were alone with her than in both conditions when the owner was present (EM means, cAO-cSO: P = 0.009, cAO-cEO: P≤0.001) and there was a non-significant trend that they spent more time close to her when the owner was replaced (EM means, cAO-cRO: P = 0.089). Further, the dogs also spent more time close to the experimenter when the owner was replaced than when the owner was present and encouraging (EM means, cRO-cEO: P = 0.016). There were no significant differences between the other conditions (EM means, cRO-cSO: P = 0.369, cSO-cEO: P = 0.110). Neither the sequence nor the type of apparatus, nor any of the interactions had an effect on how long the dogs continued to manipulate the apparatus (full results of the LMM can be seen in File S1)

### Discussion

In Experiment 2 we replicated our findings from Experiment 1 and showed that the dogs manipulated the apparatuses for shorter durations when they were alone with the experimenter than when the owner was present. When the owner was replaced with an unfamiliar human then dogs manipulated for shorter periods than when the owner was encouraging them. The difference in the duration of manipulation when the owner was present and silent and when the unfamiliar human was present was not significant. Importantly, the presence of the unfamiliar human did not significantly increase the dogs' manipulation compared when they were alone with the experimenter. Furthermore, the dogs spent significantly more time in the proximity of the owner than of the unfamiliar human. As in Experiment 1, the dogs spent most time in the proximity of the experimenter when they were alone with her and they spent more time close to her when the owner was replaced by an unfamiliar human than when the owner was present and encouraging.

By including the control condition “Replaced owner” in this experiment we could show that the owner had a specific effect on the dog's behavior in this manipulative problem-solving task. The fact that the presence of an unfamiliar human did not significantly increase the duration of manipulation in the dogs compared to when they were alone with the experimenter provides evidence for a secure base effect in dogs that is specific for the owner and therefore comparable to the one found in infant-caregiver relationships [4]. While the dogs also manipulated the apparatus significantly shorter when the unfamiliar human was present than when their owner was encouraging them, the difference in the duration of manipulation in the presence of the silent owner and the unfamiliar human was not significant. The smaller difference between those two conditions can be attributed to the fact that the dogs manipulated the apparatuses for somewhat – albeit not significantly – shorter durations when the owner was silent than when they were encouraging (see Figure 5). In our experiment we asked the owners to wear opaque sunglasses and not to react in any way to their dogs in the condition “Silent owner”. It is possible that the dogs perceived this behavior as unnatural. In human children a comparable situation (i.e., the still-face paradigm: after an initial social interaction, the adult suddenly becomes unresponsive) evokes negative affect, confusion and attempts to re-establish reciprocity with the interaction partner [14], [15]. In line with that, the dogs spent a considerable amount of time in the proximity of the owner in this situation, often in close physical contact. To avoid this effect it might have been better to allow the owners to interact naturally with their dogs while instructing them not to encourage the dogs. Interestingly, the dogs spent significantly more time in the proximity of their owners when they were silent compared to the equally silent unfamiliar human. This indicates that the lack of interaction on the part of the unfamiliar human did not evoke the same negative affect and attempts to establish proximity in the dogs. As a follow-up to this study, it might also be interesting to investigate how the interaction with and encouragement coming from the unfamiliar human would influence the dogs' motivation to manipulate.

In this experiment the dogs also differentiated between the apparatuses and there was a preference to manipulate one of them longer than the others, which was not the case for the dogs tested in Experiment 1. Before testing the dogs in both experiments the owners confirmed that the toys used as apparatuses were unknown to their dogs. However, more owners from the second sample of dogs, which was tested in Austria, reported that they occasionally provided their dogs with the type of toy, which had to be manipulated to receive a food reward. Therefore, it is possible that the varying manipulation durations in Experiment 2 were due to the resemblance of this specific toy to toys that the dogs already knew. However, despite of these differences between the toys, the pattern of manipulation in the four conditions was still evident.

As in Experiment 1 we found that the dogs spent most time close to the experimenter when they were alone with her and some more time when the owner was replaced by an unfamiliar human. This again indicates that she acted as a source of social support for the dogs.

## General Discussion

Our study provides an important piece of evidence for the similarity between the secure base effect found in dog-owner and infant-caregiver relationships. Further, our study is the first to show that the secure base effect in dogs extends from the ASST [12] to other areas of dogs' lives and that it can also manifest in cognitive testing. A comparable effect has been shown in human children when they were confronted with a problem-solving task: those children that were able to use their mother as a secure base were found to be more motivated and persistent in solving the task [13]. However, while the secure base effect is usually only evident in infanthood, where it balances the infants' exploration of the world with maintaining the crucial proximity to the caregiver [4], dogs seem to be unique in having retained this behavior into adulthood. Dogs living in animal shelters have even been found to establish preferences for specific humans after short positive interactions in adulthood, which already strikingly resemble attachment bonds [16].

Although the secure base effect we found in this study was specific for the owner, unfamiliar humans like the experimenter also seem to be able to provide some social support for the dogs. This is also suggested by the fact that the replacement of the owner with an unfamiliar human slightly – albeit not significantly – increased manipulation in the dogs. A similar effect has been observed in human children in the ASST when they seek social support from non-attachment figures with whom they had been familiarized prior to the test [17]. Although in adult dogs it has so far mainly been shown that owners are the ones who provide social support for their dogs [18], [19], in dog puppies social support can also be provided by an unfamiliar human [20].

Finally, our results also have important implications for behavioral testing in dogs. Although in our task the dogs did not need to apply sophisticated problem-solving skills and we were interested in their general motivation to manipulate the toys and not in their success, it is likely that the presence or absence of the owner might also substantially influence dogs' motivation in other more complex test situations. The owner's absence in the generally unfamiliar experimental setting might cause a lack of security, which in turn could influence the outcome of the test.

## Supporting Information

File S1 **Supplementary material.** Supplementary methods for Ainsworths's Strange Situation Test (ASST) carried out prior to Experiment 1 and full results of the Linear Mixed Models (LMM) calculated in Experiment 1 and 2.
(PDF)

Movie S1 **Condition “Absent owner”.** This video clip shows the beginning of a test trial in the condition “Absent owner” with the apparatus Hunter© Rolling Snack. Neither the owner nor the unfamiliar person is present. The experimenter releases the dog and then takes her position. The duration of the full trial is 5 minutes.
(MP4)

Movie S2 **Condition “Silent owner”.** This video clip shows the beginning of a test trial in the condition “Silent owner” with the apparatus Hunter© Snack Bottle. The owner (sitting on the chair) is present, wears opaque sunglasses and remains silent. The experimenter releases the dog and then takes her position. The duration of the full trial is 5 minutes.
(MP4)

Movie S3 **Condition “Encouraging owner”.** This video clip shows the beginning of a test trial in the condition “Encouraging owner” with the apparatus Nina Ottosson© Dog Pyramid. The owner (sitting on the chair) is present, wears normal sunglasses and is allowed to encourage the dog. The experimenter releases the dog and then takes her position. The duration of the full trial is 5 minutes.
(MP4)

Movie S4 **Condition “Replaced owner”.** This video clip shows the beginning of a test trial in the condition “Replaced owner” with the apparatus Hunter© Snack Cactus. The unfamiliar person (sitting on the chair) is present, wears opaque sunglasses and remains silent. The experimenter releases the dog and then takes her position. The duration of the full trial is 5 minutes.
(MP4)
