# Supplementary material for: The Importance of the Secure Base Effect for Domestic Dogs – Evidence from a Manipulative Problem-Solving Task
Source: PLoS One. 2013 May 29;8(5):e65296. doi: 10.1371/journal.pone.0065296 (PMC3667003; doi:10.1371/journal.pone.0065296)
Supplement: File S1 — Supplementary material. Supplementary methods for Ainsworths's Strange Situation Test (ASST) carried out prior to Experiment 1 and full results of the Linear Mixed Models (LMM) calculated in Experiment 1 and 2. (PDF) [file pone.0065296.s001.pdf]

Supplementary material for:

"The Importance of the Secure Base Effect for Domestic Dogs – Evidence from a Manipulative Problem-Solving Task"

Supplementary methods

Ainsworth's Strange Situation Test (ASST)

We used a shortened version of the original ASST [S1] modified to be applicable for testing dogs. The ASST can be used for measuring the different components of dogs' attachment to their owners (i.e. proximity maintenance, separation distress, secure base, and safe haven effects). For the purpose of the current study, we were only interested in dogs' display of separation-related behaviors (SRB; [S2]) when left alone, indicating their degree of separation anxiety in the absence of the owner.

Dogs were tested in the ASST 1-18 weeks (mean±SD=6.7±5.93) prior to the main experiment. The ASST took place in a different experimental room (3.5m x 4.5m) that was unknown to the dogs. The room contained two chairs (one chair for the owner and one chair for the stranger), two shelves, building blocks placed on one shelf out of the dog's reach, several toys placed on the floor, and a water bowl with fresh water.

The ASST consisted of seven episodes, each lasting approximately 3 minutes. In three episodes a stranger was present in the room. The stranger was of the same sex as the dog owner and had never been seen by or interacted with the dog prior to the experiment.

- *Episode 1: Dog with owner*

The owner entered the experimental room with the dog on leash, sat down on the designated chair, took the leash off and let the dog run free. The owner put the leash down beside the chair. During the first 2 minutes the owner filled out a questionnaire without interacting with the dog. After hearing a signal from outside, the owner carried building blocks from one shelf to the other for 1 minute without interacting with the dog. After that, the owner sat down and continued filling out the questionnaire without interacting with the dog.

- *Episode 2: Dog with owner and stranger*

The stranger entered the room and sat down on the other chair passively for 1 minute. Then the stranger tried to engage the dog to play for 2 minutes. After the first minute, the stranger asked the owner to leave the room.

- *Episode 3: Dog with stranger*

The stranger sat down the designated chair. During the first 2 minutes the stranger filled out a questionnaire without interacting with the dog. After that, the stranger carried building blocks from one shelf to the other for 1 minute without interacting with the dog. After that, the stranger left the room.

- *Episode 4: Dog alone*

The dog stayed alone in the room for three minutes.

- *Episode 5: Dog with owner*

The owner entered the room. After shortly greeting the dog, the owner sat down on the designated chair and filled out a questionnaire for 3 minutes without interacting with the dog. After hearing a signal from outside, the owner left the room.

- *Episode 6: Dog alone*

The dog stayed alone in the room for three minutes.

- *Episode 7: Dog with stranger*

The stranger entered the room. After shortly greeting the dog, the stranger sat down on the designated chair and filled out a questionnaire for 3 minutes without interacting with the dog. After that, the stranger took the dog on the leash and left the room together with the dog.

During the two episodes, in which the dog was left alone in the room (Episode 4, Episode 6), we scored dogs' SRB according to Mendl et al. [S3]. SRB comprised vocalizing (barking, whining, howling), staying close to the door (with or without scratching the door), destructive behavior, and defecation/urination. Since destructive behavior and defecation/urination were never observed during the two episodes, those behaviors were excluded from the analysis. A score of 0 was given for each of the two behaviors (i.e. vocalizing, staying close to the door), if the behavior occurred never or less than 25% of time during the episode, a score of 1 was given, if the behavior occurred between 25% and 50% of the time, and a score of 2 was given, if the behavior occurred more than 50% of the time. The scores from both behaviors were added to give the total SRB score.

Supplementary results

Linear mixed models (LMM)

Effects of the main factors factors "sequence of conditions" (1st, 2nd, 3rd), "type of apparatus" (aDP, aSB, aSC, aRS), and "condition" (cAO, cSO, cEO) and the two-way interactions on the variable "duration of manipulation" as calculated by an LMM can be seen in Table S1.

Table S1. Effects on the variable "duration of manipulation".

Dependent variable	N	df_{NUM}	df_{DEN}	Factor	F	P
Duration of manipulation	20	2	36	*Sequence of conditions*	1.522	0.232
		3	36	*Type of apparatus*	0.464	0.709
		2	36	*Condition*	12.478	0.000***
		6	36	*Sequence*Apparatus*	1.161	0.348
		4	36	*Sequence*Condition*	0.565	0.690
		6	36	*Apparatus*Condition*	1.172	0.343

Effects of the main factors factors "sequence of conditions" (1st, 2nd, 3rd), "type of apparatus" (aDP, aSB, aSC, aRS), and "condition" (cSO, cEO) and the two-way interactions on the variable "duration spent in the proximity of the owner" as calculated by an LMM can be seen in Table S2.

Table S2. Effects on the variable "duration spent in the proximity of the owner".

Dependent variable	N	df_{NUM}	df_{DEN}	Factor	F	P
Duration spent in the proximity of the owner	20	2	22	*Sequence of conditions*	0.242	0.787
		3	22	*Type of apparatus*	1.092	0.373
		1	22	*Condition*	0.017	0.897
		6	22	*Sequence*Apparatus*	0.756	0.612
		2	22	*Sequence*Condition*	2.198	0.135
		3	22	*Apparatus*Condition*	1.179	0.341

Effects of the main factors factors "sequence of conditions" (1st, 2nd, 3rd), "type of apparatus" (aDP, aSB, aSC, aRS), and "condition" (cAO, cSO, cEO) and the two-way interactions on the variable "duration spent in the proximity of the experimenter" as calculated by an LMM can be seen in Table S3.

Table S3. Effects on the variable "duration spent in the proximity of the experimenter".

Dependent variable	N	df_{NUM}	df_{DEN}	Factor	F	P
Duration spent in the proximity of the experimenter	20	2	36	*Sequence of conditions*	4.611	0.016*
		3	36	*Type of apparatus*	0.488	0.720
		2	36	*Condition*	17.221	0.000***
		6	36	*Sequence*Apparatus*	1.489	0.210
		4	36	*Sequence*Condition*	2.923	0.034*
		6	36	*Apparatus*Condition*	1.104	0.379

Effects of the main factors factors "sequence of conditions" (1st, 2nd, 3rd, 4th), "type of apparatus" (aDP, aSB, aSC, aRS), and "condition" (cAO, cRO, cSO, cEO) and the two-way interactions on the variable "duration of manipulation" as calculated by an LMM can be seen in Table S4.

Table S4. Effects on the variable "duration of manipulation".

Dependent variable	N	df_{NUM}	df_{DEN}	Factor	F	P
Duration of manipulation	26	3	67	*Sequence of conditions*	0.798	0.499
		3	67	*Type of apparatus*	5.509	0.002**
		3	67	*Condition*	7.700	0.000***
		9	67	*Sequence*Apparatus*	0.593	0.798
		9	67	*Sequence*Condition*	0.770	0.644
		9	67	*Apparatus*Condition*	0.793	0.624

Effects of the main factors factors "sequence of conditions" (1st, 2nd, 3rd, 4th), "type of apparatus" (aDP, aSB, aSC, aRS), and "condition" (cAO, cRO, cSO, cEO) and the two-way interactions on the variable "duration spent in the proximity of the experimenter" as calculated by an LMM can be seen in Table S5.

Table S5. Effects on the variable "duration spent in the proximity of the experimenter".

Dependent variable	N	df_{NUM}	df_{DEN}	Factor	F	P
Duration spent in the proximity of the experimenter	26	3	67	*Sequence of conditions*	0.060	0.981
		3	67	*Type of apparatus*	0.609	0.611
		3	67	*Condition*	8.257	0.000***
		9	67	*Sequence*Apparatus*	1.421	0.197
		9	67	*Sequence*Condition*	0.620	0.776
		9	67	*Apparatus*Condition*	0.961	0.479

Supplementary references

S1. Ainsworth MDS, Wittig BA (1969) Attachment and exploratory behavior of one-year olds in a strange situation. In: Foss BM, editor. Determinants of infant behavior (Vol. 4). London: Methuen. pp. 111-136.

S2. Bradshaw JWS, McPherson JA, Casey RA, Larter IS (2002) Aetiology of separation-related behaviour in domestic dogs. Vet Rec 151:43–36. doi:10.1136/vr.151.2.43.

S3. Mendl M, Brooks J, Basse C, Burman O, Paul E, Blackwell E, Casey R (2010) Dogs showing separation-related behaviour exhibit a 'pessimistic' cognitive bias. Curr Biol 20:R839-R840. doi:10.1016/j.cub.2010.08.030.
